# Identification of a conserved *var* gene in different *Plasmodium falciparum* strains

**DOI:** 10.1186/s12936-020-03257-x

**Published:** 2020-05-29

**Authors:** Sandra Dimonte, Ellen I. Bruske, Corinna Enderes, Thomas D. Otto, Louise Turner, Peter Kremsner, Matthias Frank

**Affiliations:** 1 Institute of Tropical Medicine, University of Tuebingen, Wilhelmstr. 27, 72074 Tuebingen, Germany; 2 Malaria Programme, Wellcome Trust Sanger Institute, Hinxton, CB10 1SA UK; 3 Centre of Immunobiology, Institute of Infection, Immunity & Inflammation, College of MVLS, University of Glasgow, Glasgow, UK; 4 Centre for Medical Parasitology, Department of Immunology and Microbiology (ISIM), Faculty of Health and Medical Sciences, University of Copenhagen, 1165 Copenhagen, Denmark; 5 Department of Infectious Diseases, Copenhagen University Hospital (Rigshospitalet), 2100 Copenhagen, Denmark

**Keywords:** Malaria, *var* genes, Genetic diversity, Microsatellites, Recombination, VSA, PfEMP1, *rifin*, *stevor*

## Abstract

**Background:**

The multicopy *var* gene family of *Plasmodium falciparum* is of crucial importance for pathogenesis and antigenic variation. So far only *var2csa,* the *var* gene responsible for placental malaria, was found to be highly conserved among all *P. falciparum* strains. Here, a new conserved 3D7 *var* gene (PF3D7_0617400) is identified in several field isolates.

**Methods:**

DNA sequencing, transcriptional analysis, Cluster of Differentiation (CD) 36-receptor binding, indirect immunofluorescence with PF3D7_0617400-antibodies and quantification of surface reactivity against semi-immune sera were used to characterize an NF54 clone and a Gabonese field isolate clone (MOA C3) transcribing the gene. A population of 714 whole genome sequenced parasites was analysed to characterize the conservation of the locus in African and Asian isolates. The genetic diversity of two *var2csa* fragments was compared with the genetic diversity of 57 microsatellites fragments in field isolates.

**Results:**

PFGA01_060022400 was identified in a Gabonese parasite isolate (MOA) from a chronic infection and found to be 99% identical with PF3D7_0617400 of the 3D7 genome strain. Transcriptional analysis and immunofluorescence showed expression of the gene in an NF54 and a MOA clone but CD36 binding assays and surface reactivity to semi-immune sera differed markedly in the two clones. Long-read Pacific bioscience whole genome sequencing showed that PFGA01_060022400 is located in the internal cluster of chromosome 6. The full length PFGA01_060022400 was detected in 36 of 714 *P. falciparum* isolates and 500 bp fragments were identified in more than 100 isolates. *var2csa* was in parts highly conserved (H_e_ = 0) but in other parts as variable (H_e_ = 0.86) as the 57 microsatellites markers (H_e_ = 0.8).

**Conclusions:**

Individual *var* gene sequences exhibit conservation in the global parasite population suggesting that purifying selection may limit overall genetic diversity of some *var* genes. Notably, field and laboratory isolates expressing the same *var* gene exhibit markedly different phenotypes.

## Background

The most virulent form of malaria is caused by *Plasmodium falciparum* [1]. The virulence of *P. falciparum* is a consequence of adhesion of infected red blood cells (iRBCs) to different host endothelial receptors [2]. This process is primarily mediated by a polymorphic protein family collectively referred to as *P. falciparum* membrane protein 1 (PfEMP1) [2–4]. Expression of PfEMP1 variants that exhibit binding to chondroitin sulfate [5, 6] in the placenta or to the endothelial protein C receptor (EPCR) [7] has been associated with the development of placental malaria and cerebral malaria, respectively. PfEMP1 is encoded by the *var* gene family [8], a multicopy gene family with approximately 59–60 different genes per parasite genome [9]. Only one *var* gene is expressed in an individual parasite at a time, a process referred to as mutually exclusive expression [10]. Switching between the active *var* locus generates antigenic variation leading to immune escape of the parasites [11].

*var* genes can be grouped according to their distribution across the 14 *P. falciparum* chromosomes into subtelomeric or central and this position correlates with their promoter type [12]. 36 of the 59 *var* genes in the 3D7 genome strain are located in subtelomeric areas and have upstream (Ups)A, UpsB or UpsB/A type promoters, while 23 are located in central *var* clusters and have UpsC or UpsB/C type promoters. An individual *var* gene typically consists of exon 1 and exon 2 that encode for the extracellular and intracellular parts of the PfEMP1 protein, respectively. Exon 1 is hypervariable and consists of an N-terminal segment, followed by a variable number of domain cassettes (DCs) that are composed of several Duffy Binding Like- (DBL) and Cysteine Rich Interdomain Region-(CIDR) domains [9]. DBL and CIDR domains are assembled of conserved sequence blocks that share similarities in all parasite strains as well as hypervariable sequence blocks [13]. The 60 *var* genes of an individual parasite are almost completely distinct from each other and different *P. falciparum* strains carry almost completely different *var* gene repertoires [14, 15]. This tremendous genetic diversity appears to be the result of the highly recombinogenic nature of the AT-rich *P. falciparum* genome, the diversifying pressure of the immune system and the virtual absence of purifying selection on the *var* gene family. Indeed recombination events generate new *var* gene variants during meiosis and mitosis [16, 17]. Furthermore binding phenotypes such as CD36 and EPCR binding appear to be primarily mediated by the tertiary structures of the CIDR domain and by hydrophobicity, characteristics that do not require an exact amino acid motif, thus allowing a high degree of sequence variation without affecting the function of the respective PfEMP1 domains [18].

Considering the high variability of the *var* gene family, it is remarkable that there is one *var* gene that is conserved among all *P. falciparum* strains. This gene, *var2csa*, [5] encodes the PfEMP1 VAR2CSA that binds to chondroitin sulfate A (CSA) in the human placenta and is primarily responsible for placental malaria during the first pregnancy [6]. Because of this unique binding phenotype and function, there appears to be positive selection pressure to maintain this *var* gene.

During a *var* gene transcription analysis of a field isolate from Gabon (called MOA), a DBL with 100% sequence identity to the DBL of PF3D7_0617400 of the 3D7 reference genome [19] was identified. In this work, the MOA allele of the gene (PFGA01_060022400) is shown to be 99% identical with PF3D7_0617400. The gene was detected in 36 of 714 African and Asian parasite suggesting a selective advantage for parasites carrying this *var* gene.

## Methods

### Parasite lines and cultures

The MOA bulk culture was originally obtained from an chronic asymptomatic infection of a Gabonese individual as previously described [19, 20]. A total of 19 clones were generated by limiting dilution in two independent cloning experiments [19, 20]. The MOA C3 clone and the MOA D2 (PfGB01) [21] clone were generated in the first cloning experiment directly after tissue culture adaptation of the MOA bulk culture [19]. The NF54 A3 clone was generated from an NF54 bulk culture as described previously [22]. Parasite stocks in glycerolite of the NF54 A3 [22] laboratory strain, the culture adapted Gabonese MOA C3 [19] and Δ MOA D2 (PFGB01) [20] field isolates were thawed by slowly adding 5 drops of 12% NaCl solution and a 5 ml of 1.6% NaCl solution. The stocks were then spun down, the supernatant was discarded and the parasites were used to inoculate an in vitro culture.

The isolates 5259, 12295, 5420, 12480, 5798, 3256, 3324, 6022, 6210 were culture adapted from diagnostic specimens submitted for routine malaria diagnosis to the laboratory of the outpatient clinic of the Institute of Tropical Medicine in Tübingen, Germany. After conducting routine thick and thin blood smears, the remainder of the EDTA blood tube was centrifuged, the serum was separated and 500 µl erythrocyte pellet was used to inoculate 5 ml culture medium. After successful culture adaptation, parasites were expanded into a 20 ml culture and cryopreserved stocks as well as cell pellets were stored for future investigations. The Δ MOA D2 parasite line has been previously described [20]. All *P. falciparum* isolates were cultivated at 5% haematocrit of 0^+^ erythrocytes from a local cell bank. RPMI 1640 medium was completed with 10% Albumax (Gibco), 25 mM HEPES Buffer, 2 mM l-Glutamine and 0.05 mg/ml gentamicin (all PAA Laboratories). Parasites were incubated at 37 °C in 90% nitrogen, 5% oxygen and 5% carbon dioxide. Δ MOA D2 was cultured in medium without blasticidin as previously described [20].

### DNA extraction

Cell pellets for DNA extraction were stored at − 20 °C. After thawing of the pellet, DNA was extracted using the QIAmp^®^ DNA Blood Midi Kit (Quiagen, Cat. No. 51185) following the manufacturer’s protocol. DNA content was measured by Nanodrop^®^ 1000 3.7.1 (Nanodrop Technologies).

### Polymerase chain reaction (PCR) and agarose gel electrophoresis

For qualitative PF3D7_0617400 PCR, a specific primer set was designed, spanning exon 1 from promoter until the intron (Additional file 1: Table S1). Overlapping fragments for the entire exon 1 were generated. PCR with the *var2csa* specific primers 10F and 75 R [5] was employed to amplify an approximately 1700 bp fragment of *var2csa*. The first 40 BP of exon 1 of PF3D7_0617400 were characterized with the UpsC promoter primer. This primer was originally developed by Rottmann et al. [23] and has been previously evaluated on the MOA field isolate [19].

5 µl genomic DNA were mixed with 31 µl H2O, 0.4 µl dNTPs, 3 µl MgCl, 5 µl buffer, 2.5 µl forward primer, 2.5 µl reverse primer, and 0.3 µl Taq-polymerase to a final volume of 50 µl per reaction. Standard PCR conditions were 94 °C for 3 min, 40× (94 °C for 10 s., 54 °C for 30 s., 72 °C for 30 s.), 72 °C for 3 min. For the primers pairs P3, P5 and P6 annealing and elongation temperatures were adjusted according to primer melting temperature and fragment length. Individual primer specific PCR conditions can be obtained from the author upon request. PCR fragments were separated using gel electrophoresis with a 1% agarose gel, 80–120 V current.

### Sorbitol synchronization, RNA extraction and cDNA synthesis

20 ml parasite cultures were used for RNA extraction. The culture was pelleted, washed several times with 1× PBS and the erythrocytes were lysed with 0.02% saponin. The pellet was then washed 3 times with 1× PBS and resolved in 750 µl of Trizol^®^ LS Reagent (Invitrogen). The samples were stored at − 20 °C until further processing.

For RNA extraction, 0.2 ml chloroform were added to 750 µl of thawed Trizol-lysate, shaken vigorously for 15 s and left at room temperature for 10 min. A 15 min centrifugation step at 12,000*g* at room temperature followed for phase separation. RNA was extracted from the aqueous phase with the PureLink™ RNA Mini Kit (ambion by Life Technologies) according to the manufacturer’s protocol. RNA content was measured by Nanodrop^®^ 1000 3.7.1 (Nanodrop Technologies). All samples were treated with DNAse I^®^ (Invitrogen) according to the manufacturer´s protocol to remove any remaining DNA. cDNA was synthesized with random primers and Superscript II Reverse Transcriptase^®^ (Invitrogen) according to the manufacturer´s protocol. cDNA was tested for absence of DNA contamination by evaluation of proper splicing of the gene PFD1155w by PCR with the primer 5′GCAGGGAAAGGTTTTTCAAG3′ and the reverse primer 5′AAAGCTGAATCTTGGCCCGTT 3′ as described elsewhere [22].

### *Var* gene transcription analysis

### cDNA DBL cloning

For analysis of specific *var* gene expression in the MOAC3 clone, the active *var* locus was identified by cloning cDNA *var* PCR fragments obtained with universal primers [24] followed by sequencing. The experiment was performed in two biological replicates directly after the original limiting dilution experiment of the MOA bulk culture [19].

#### Quantitative real-time PCR

For quantitative RT-PCR reactions of the NF54 A3 strain, we employed the primer set of Salanti et al. [5] with the modifications as previously described [10, 19, 25] . For quantitative RT-PCR of the MOA field isolate the MOA primers C3_C3, D2_D2 and D5_D5 were used [19]. All reactions included five housekeeping genes as controls: seryl-tRNA synthetase (PF3D7_0717700), fructose bisphosphate aldolase (PF3D7_1444800), actin (PF3D7_1246200), arginyl-tRNA synthetase (PF3D7_1218600) and glutaminyl-tRNA synthetase (PF3D7_1331700) [25]. Reactions were performed at a final primer concentration of 0.25 µM using SensiMix SYBR No-ROX Kit (Bioline, QT650-05) in 20 µl reactions, measured in Corbett Research Rotorgene 3000 (95 °C for 3 min/95 °C for 15 s, 54 °C for 30 s, 68 °C for 30 s, 40 cycles/68 °C for 1 min). The same threshold was used for all analysis. *var* gene copy numbers were determined relatively to PF3D7_1218600, using the ΔΔCT method [5].

### Targeted Sanger sequencing

For PCR fragment-sequencing, the PCR product was purified from an agarose gel or the PCR solution, using the Quiagen PCR purification kit according to the manufacturer’s protocol. Sequencing PCR was performed at a final reaction volume of 10 µl per reaction, containing 1µ BigDye, 2 µl reaction buffer, 2.5 µl forward or reverse primer, purified water and purified PCR product according to DNA concentration. Sequencing PCR conditions were: 94 °C for 10 s, 50 °C for 5 s, 60 °C for 4 min, back cycle to beginning: 25×. After PCR, all samples were cleaned up by Sephadex agarose column centrifugation. Sanger sequencing was performed in the Applied Biosystems ABI Prism 3130xl Genetic Analyzer.

### Whole genome sequencing

Long-read Pacific Biosciences whole genome sequencing of the MOA D2 (PFGB01) and 5798 (PFTG01) parasite lines parasite was performed as previously described [21].

### Fragment analysis

57 microsatellites (MS) distributed over the 14 chromosomes of *P. falciparum* were analysed by multiplex fragment analysis (Additional file 2: Table S2). DNA of all freshly culture adapted field isolates, the MOA C3 strain as well as the NF54 A3 were analysed. MS amplification and fragment length analysis were done as previously described [26]. In four strains, approximately 30% of MS showed 2 amplification products/peaks (5798, 3324, 5420, 12480). The higher peak was arbitrarily chosen as the representative allele for the respective marker.

### Calculation of expected heterozygosity

The expected heterozygosity (H_e_) was calculated using the formula H_e_ = [n/(n − 1)][1 − Ʃpi2], where n is the total number of alleles at a distinct locus and reflects the proportion of the individual alleles as described previously [27]. A new allele was defined as a difference of more > 3 bp between PCR fragments [26]. The same criterion was applied for allele definition of the *var2csa* fragments used for the H_e_ calculation of the *var2csa* gene.

### Assembling of sequences

Sequences were assembled and aligned using SeqScape software by Applied Biosystems.

### Comparing sequences

The long read sequences were compared using the ACT software by Artemis [28]. To compare the *var* gene sequences with the *var* gene database, a megablast (-e 1e-6 -m 8 -a 8 -v 15000 -b 15000 -F F) against the normalised data base from Otto et al. [15] was done (varDB.Normalised.3 kb.nt.noVARx_noExon2.fasta.gz)–ftp ftp://ftp.sanger.ac.uk/pub/project/pathogens/Plasmodium/falciparum/PF3K/varDB/NormalisedDataset/. To look for SNP around the conserved locus on chromosome 6, SNP were called with mpileup : (bcftools mpileup -r Pf3D7_06_v3 -f Pf3D7_06_v3.fasta $x.bam | bcftools call -cv -Ov –ploidy 1 -o SNP.var.$x.vcf).

### Antibody preparation and Immunofluorescence assay (IFA)

The recombinant CIDRa2.1 domain of PF3D7_0617400 (MAL6P1.252) was produced at the Statens Seruminstitut, Cophenhagen, Denmark as described in [18] and used to immunize mice. Blood of immunized mice was sent to the Institute of Tropical Medicine, Tübingen, Germany. Mouse sera containing IgG α PF3D7_0617400 mouse secondary antibody were depleted from RBCs by filtering through a MN 615 ¼ filter. IgG was purified using Protein G Spin Columns from Thermo Scientific according to the manufacturer’s protocol.

For IFA, very thin blood smears of parasite cultures were fixated in − 20 °C cold 100% methanol for 5 min and then stored at − 20 °C until further use. IFA was performed following a modified protocol as previously described [29].

After a 5 min rehydration step in 1xPBS, slides were incubated for 1–2 h with anti CIDRa2.1 PF3D7_0617400 (diluted 1:50 in 1xPBS/1%BSA). The slides where then washed 3× with 1xPBS and incubated for 1 h with Alexa488 coupled mouse sera IgG α PF3D7_0617400 mouse secondary antibody (not diluted, 0.44 mg/ml). After another 3× washing with 1xPBS, slides were stained with Hoechst 33342 (diluted 1:1000) for 30 min. Slides were mounted over night with MOWIOL-488 and viewed through 100× oil immersion lens at a fluorescent microscope.

### CD36 receptor binding selection

Human melanoma C32 (ATCC1, CRL-1585™) cells were used to select CD36 binding infected red blood cells (iRBCs) as previously described [20].

### Flow cytometry analysis

Flow cytometry analysis of MOA C3, NF54 A3 and Δ MOA D2 with serum of the MOA individual was performed as described in [20].

## Results

### Identification of the 3D7 *var* gene PF3D7_0617400 in a field isolate from Gabon

**Fig. 1:**
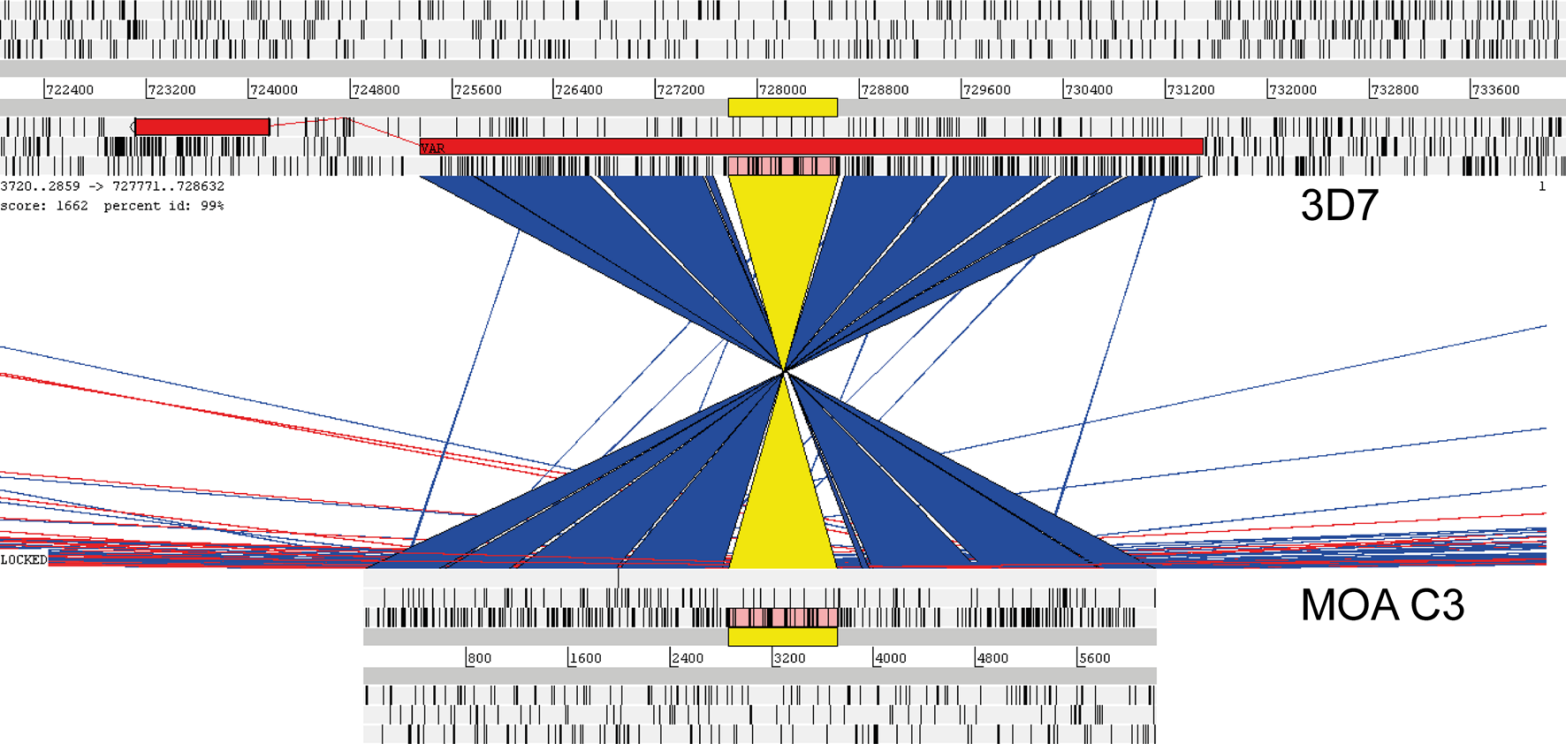
Screenshot of ACT program comparing the assembled sequence of PF3D7_0617400 of the field isolate clone MOA C3 with the reference strain 3D7. The upper part of the figure depicts the 3D7 reference sequence. The lower part shows the MOA C3 sequence. Blue and yellow = sequence identical. White: missing sequence (insertion). For orientation, the sequence next to the insertion that differs between MOAC3 and 3D7 is coloured in yellow. The additional small white areas represent homopolymer runs. Visual examination of direct alignments of the homopolymer runs revealed no differences in these areas (data not shown)

The *P. falciparum* field isolate MOA was obtained from an asymptomatically infected Gabonese individual [19, 20, 30]. After tissue culture adaption, a total of 19 clones were generated by limiting dilution and the transcribed *var* genes were determined by cDNA DBL cloning. In the MOA clone C3 [19], the transcribed DBL (MOA-C3) was found to be 100% identical with the DBL of the 3D7 *var* gene PF3D7_0617400 (previously annotated as MAL6P1.252 /PFF0845c). To determine the extent of sequence homology, a primer set (Additional file 1: Table S1) spanning the promoter region and the entire exon one (bp 0-6271) of PF3D7_0617400 was designed based on the 3D7 genome sequence. All PCR fragments obtained from MOA C3 genomic DNA were characterized by Sanger sequencing and comparison of the assembled MOA C3 sequence (GenBank accession number MG507307) with the 3D7 reference sequence showed that the sequences were 99–100% identical (Fig. 1). The only difference consisted of a small 194 bp insertion between base pairs 3722 and 3871 of the MOA C3 gene (3721 and 3914 of the 3D7 reference sequence).

### MOA C3 and NF54 A3 both express the same *var* gene but have different cytoadhesion and surface recognition signal phenotypes

**Fig. 2:**
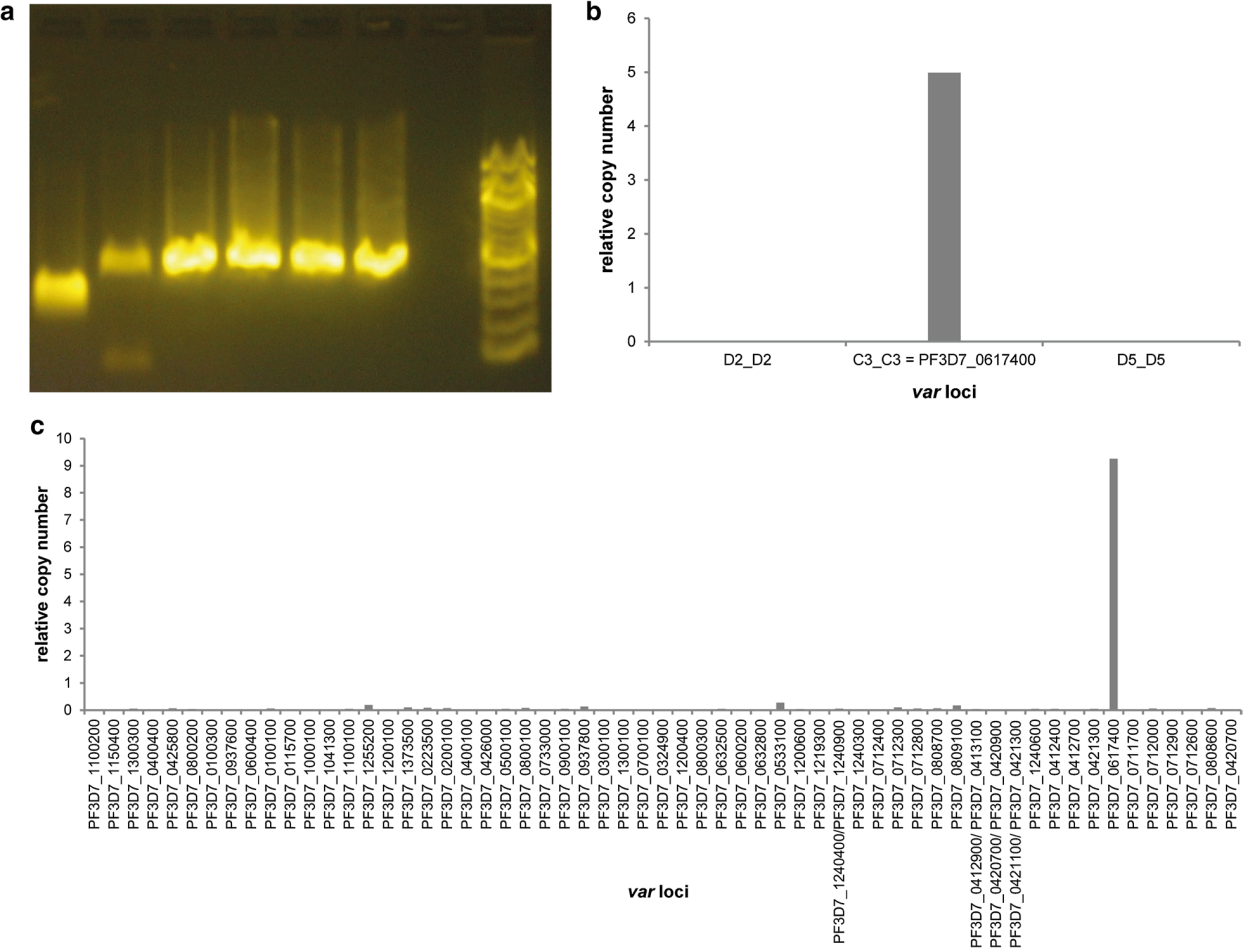
**a** Gel electrophoresis after PCR with 6 specific PF3D7_0617400 primers (primer pairs 1,2,3,4,5,6 and a 100 bp marker) on MOA C3 cDNA reveals transcription of the entire exon 1 sequence. **b** qRT-PCR with P_C3 and two other MOA DBL primers (encoding for the dominant *var* genes in the other clones generated in the same limiting dilution experiment) [19] shows that PF3D7_0617400 is transcribed as the dominant *var* gene in MOA-C3. **c** qRT-PCR with NF54 specific primer set on cDNA of NF54 A3 [22] shows continued dominant transcription of PF3D7_0617400. Transcription is quantified in relative copy numbers of the housekeeping gene arginyl tRNA synthetase in MOA C3 and NFA3

To determine if the entire open reading frame (ORF) of PF3D7_0617400 was transcribed in MOA C3, cDNA was analysed by qualitative and quantitative PCR. PCR transcripts were obtained with 6 primers spanning exon 1 of PF3D7_0617400 (Fig. 2a) showing that the full length RNA of the gene was transcribed and quantitative PCR (qPCR) with DBL specific primers showed that the transcriptional signal for PF3D7_0617400 was in the range of a dominantly transcribed *var* gene in MOA C3 (Fig. 2b). During previous investigations the NF54 clone A3 had been generated that transcribed PF3D7_0617400 in a very stable fashion [22]. NF54 A3 was therefore re-thawed and brought into tissue culture. Consistent with the previously reported low off rate of PF3D7_0617400, it was still the dominantly transcribed *var* gene in this re-grown culture (Fig. 2c).

**Fig. 3:**
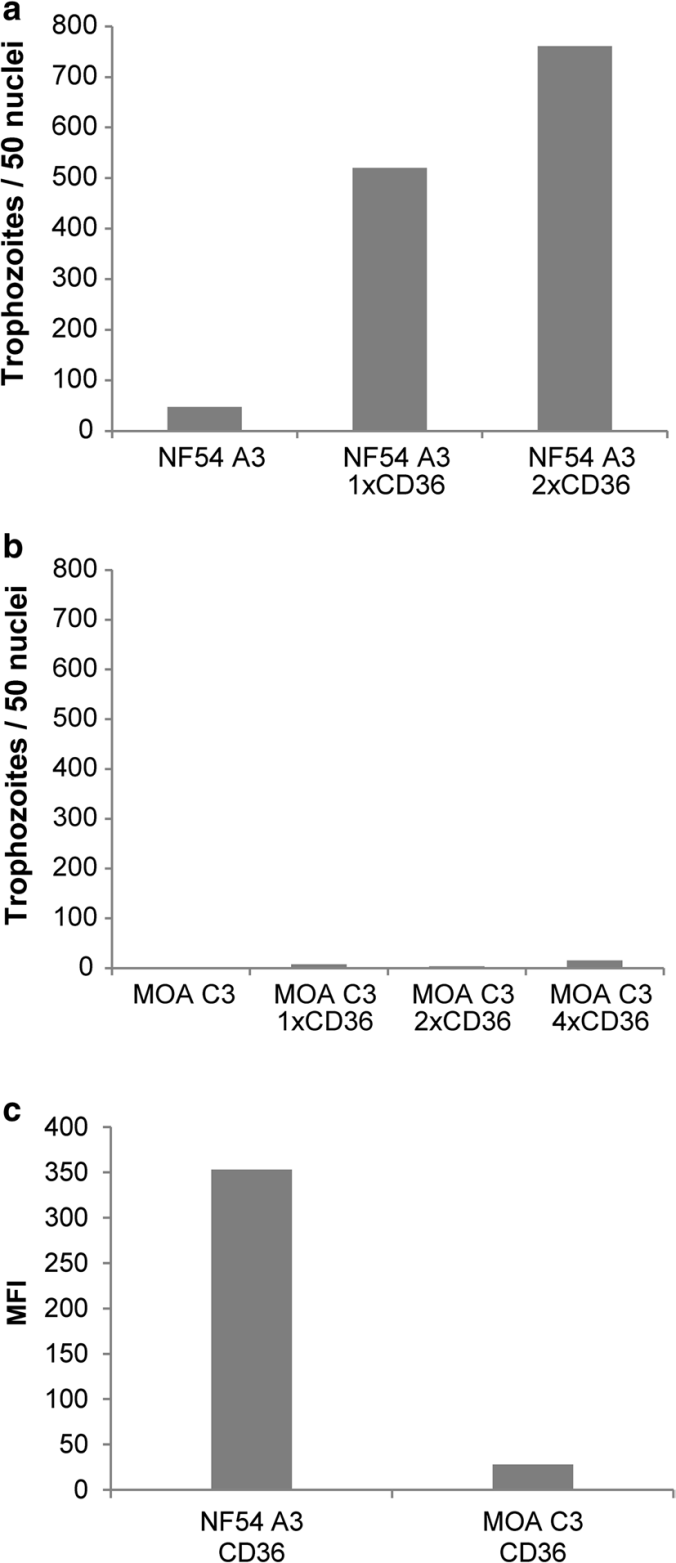
CD36 binding capacity of **a** NF45 A3 before CD36 selection and after one and two rounds of selection and **b** MOA C3 before CD36 selection as well as after one to four rounds of selection. **c** Mean fluorescence intensity (MFI) of FACS analysis with semi-immune sera from the MOA individual [20] on NF54 A3 and MOA C3 after CD36 selection

To assess the binding phenotype of PF3D7_0617400, CD36 binding assays with the clones NF54 A3 and MOA C3 were conducted on human melanoma cells expressing CD36. NF54 A3 exhibited CD36 binding even without prior selection and the CD36 binding capacity increased strongly after 2 additional rounds of panning (Fig. 3a). In contrast, MOA C3 did not exhibit significant CD36 binding prior to selection and even after 4 rounds of panning CD36 binding increased only marginally (Fig. 3b). NF54 A3 transcriptional analysis after CD36 selection showed a strong increase in PF3D7_0617400 transcription, showing that it mediated the phenotype (Additional file 3: Fig. S1a). However, transcriptional quantification of PF3D7_0617400 in MOA C3 showed no increase in transcriptional signal (Additional file 3: Fig. S1b). To quantify overall surface PfEMP1 expression in CD36 selected NF54 A3 and MOA C3 clones, FACS analysis with serum from the semi-immune MOA [20] patient was conducted. CD36 selected NF54 A3 parasites displayed a strong FACS signal with the semi-immune serum, clearly showing that the serum detected the expressed PfEMP1 on the surface of the iRBCs (Fig. 3c). In contrast, CD36 selected MOA C3 parasites only displayed a low FACS signal with semi-immune serum.

**Fig. 4:**
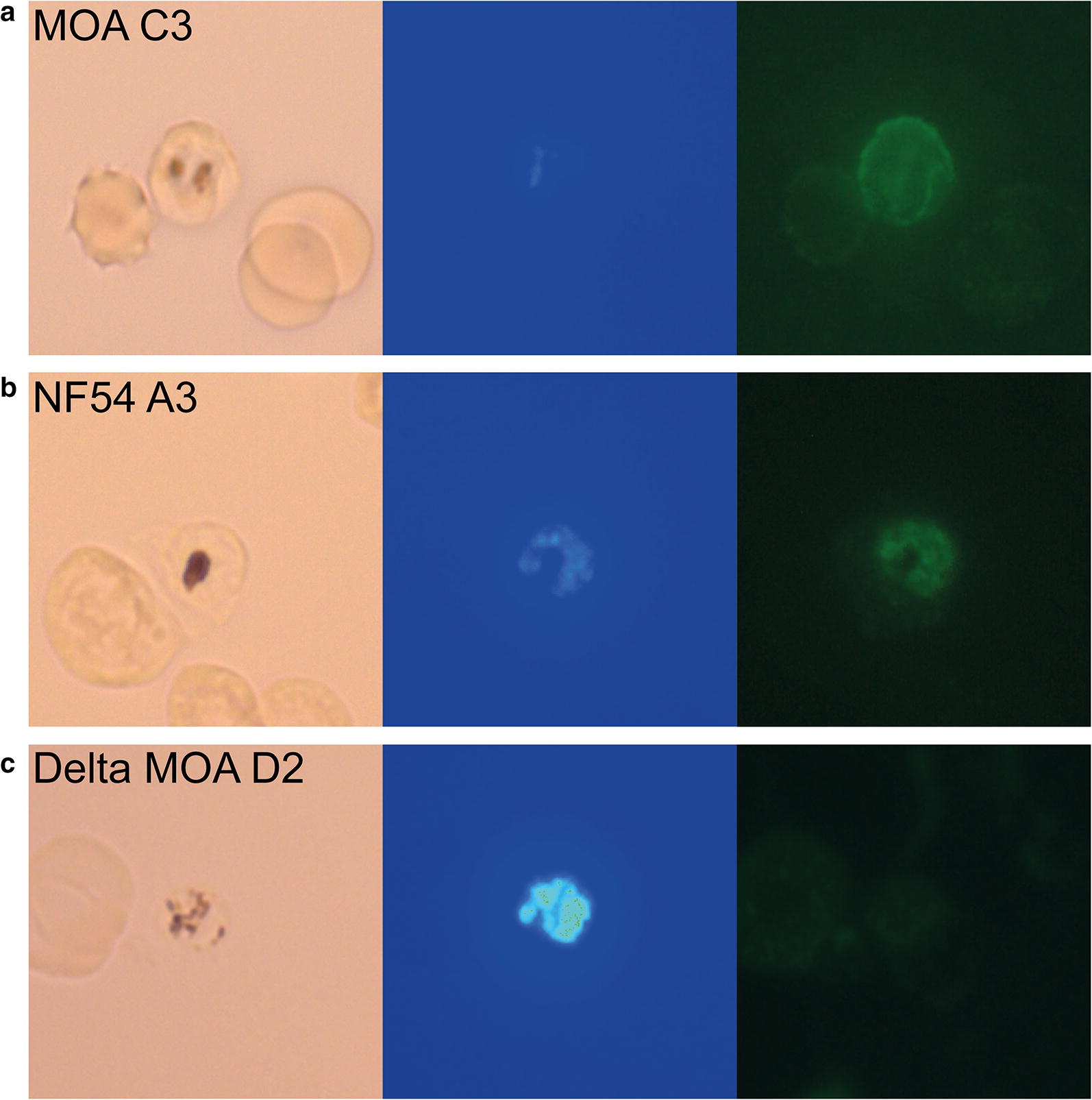
Immunofluorescence with a specific antibody against the CIDRa2.1 of PF3D7_0617400 shows expression in MOA C3 and NF54 A3 but not in the field isolate Δ MOA D2: **a** MOA C3, **b** NF54 A3, **c** Δ MOA D2 (propagated without blasticidin pressure). The first images displays light microscopy, the second image identifies parasitized RBCs by Hoechst DNA staining (blue) and the third image displays IFA with mouse anti- CIDRa2.1-PF3D7_0617400 antibody and GFP. Note the anti- CIDRa2.1 antibodies signal in MOA C3 and NF54 A3. The D2 DBL expressed in Δ MOA D2 [20] is not detected by the antibody

To determine if the corresponding PfEMP1 was indeed expressed in both strains, immunofluorescence assays with an antibody against the CIDRa2.1 of PF3D7_0617400 were conducted [18] . An immunofluorescence signal was detected in MOA C3 (Fig. 4a) and in the NF54 A3 infected erythrocytes (Fig. 4b) [20] showing that both strains indeed expressed the same PfEMP1 [20]. Together the data suggested that although both strains expressed the same *var* gene, there was a difference in PfEMP1 display between the NF54 and MOA parasites.

### Identification of PF3D7_0617400 in *P. falciparum* field isolates from West, Central and East Africa

**Fig. 5:**
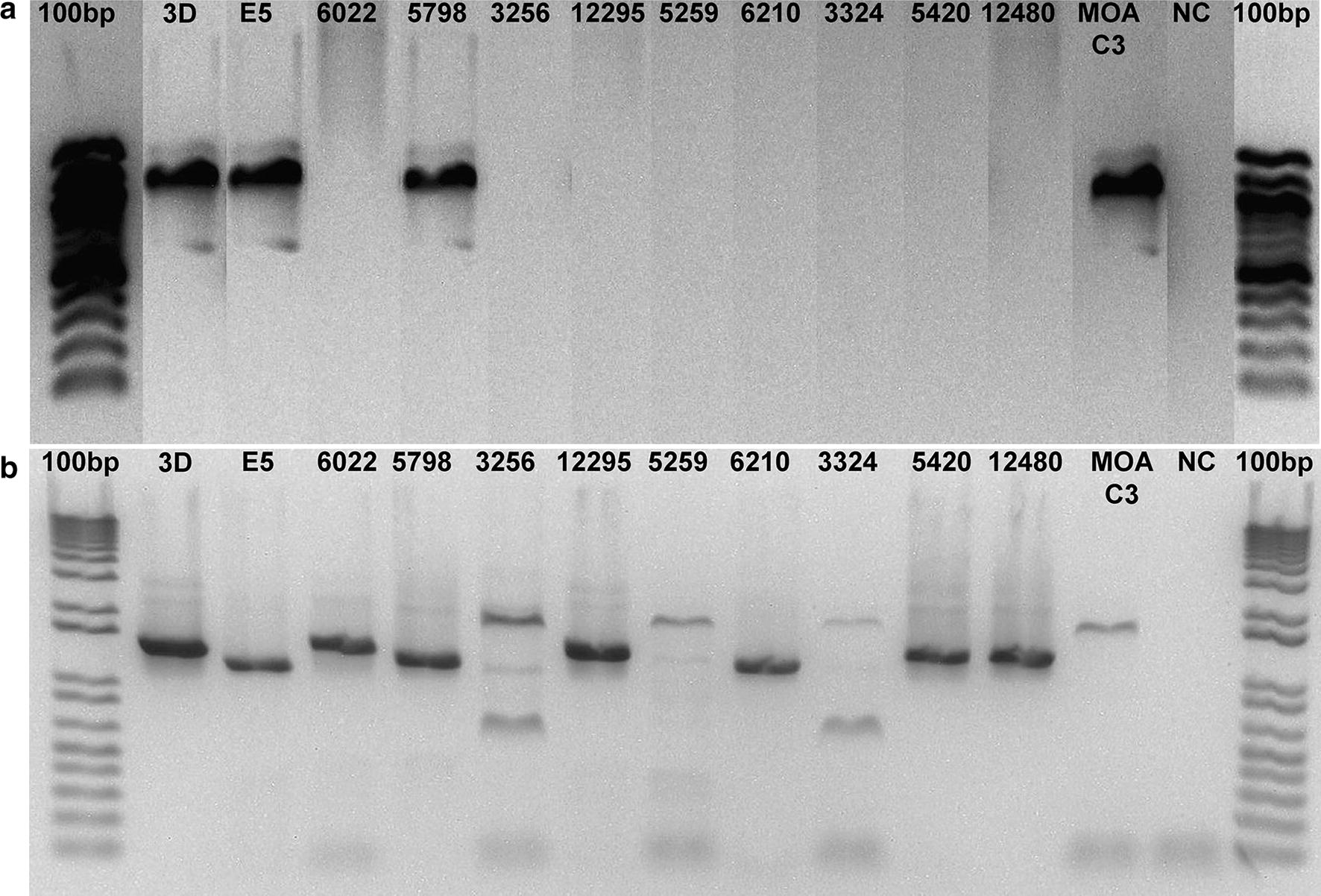
PF3D7_0617400 and *var2csa* in field isolates. **a** Gel electrophoresis of PCR fragments of PF3D7_0617400 on all field isolates and controls. PCR fragments of PF3D7_0617400 amplified (primer pair P0F + P0.1R) on two field isolated (5798, MOA C3) and on the NF54 clones 3D7 and E5 (positive controls). NC = negative control. The gel was digitally rearranged to match the gel shown in **b**. **b** Gel electrophoresis of PCR fragments of *var2csa* on all field isolates and controls. PCR fragments of *var2csa* amplified by one primer pair (10F and 75R, [5]) on all field isolates and on the NF54 clones 3D7 and E5 (positive controls). NC = negative control

**Fig. 6:**
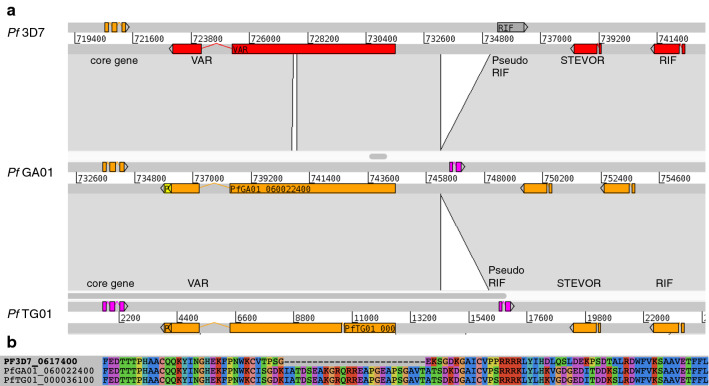
**a** ACT program comparative analysis of the central cluster of chromosome 6 in the 3D7 genome strain, MOA C3 (PFGA01) (middle part of figure) and 5798 (PFTG01) (lower part of figure), note that the *stevor* and *rifin* genes located downstream of PF3D7_0617400 are also conserved in the 3 strains. **b** Amino acid alignment depicting the amino acid sequence difference between 3D7 and the two field isolates

To generate a first estimate of the degree of PF3D7_0617400 conservation in *P. falciparum* strains, a panel of 9 additional freshly culture adapted field isolates from Central Africa (Congo, Cameroon), West Africa (Gambia, Ghana and Togo) and East Africa (Kenya and Sudan) (Table 1) were evaluated for the presence of the PF3D7_0617400 *var* locus and the highly conserved *var2csa* gene [26]. All field isolates were obtained from travellers who developed symptomatic *P. falciparum* infections after returning from Africa [21]. After successful tissue culture adaptation, DNA of the field isolates was screened by targeted PCR. Initial screening for a 3 kb fragment of PF3D7_0617400 detected the gene in a field isolate originating from Togo (5798) (Fig. 5a). Subsequent PCR with primers spanning the entire exon 1 (Additional file 1: Table S1) amplified identical fragments in 5798 and 3D7 and targeted Sanger sequencing of the field isolate 5798 (GenBank accession number MG507306) showed that it was 99–100% identical with the 3D7 reference sequence. The only difference in the exon 1 sequence was again an insertion of 194 bp ranging from 3722 to 3871 bp (3721 and 3914 of the 3D7 reference sequence) (Fig. 6).

**Table 1:** Field isolate overview: isolate numbers, country of origin

Isolate No.	Country of origin
5259	Congo
12295	Cameroon
5420	Kenya
12480	Kenya
5798	Togo
3256	Togo, Ghana
3324	Kenya
6022	The Gambia
6210	Sudan
MOA C3	Gabon

To analyse the chromosomal context of the conserved gene, Long-read Pacific Biosciences whole genome sequencing [21] of the MOA (PFGA01) and 5798 (PFTG01) parasite lines was performed. In both strains the conserved gene was located in the central cluster on chromosome 6. The field isolate allele of the MOA strain was annotated as PFGA01_060022400. Interestingly the neighbouring *stevor* and *rifin* genes were also conserved in the three isolates (Fig. 6).

The whole genome sequence analysis, as well as the microsatellite analysis (see below) of PFTG01 showed that it was a mixed infection of two different parasites strains. Consequently, the *de novo* assembly process generated two different alleles of the central cluster on chromosome 6 (as well as for all other variable parts of the genome). The allele carrying the central cluster that is identical with the PF3D7 central cluster is located on contig PFTG01_00_33.embl.

### Conservation of PF3D7_0617400 and PfGA01_060022400 in a global *P. falciparum* population from Africa and Asia

To evaluate if the presence of PF3D7_0617400/ PFGA01_060022400 in 2 out of 10 field isolates reflected the conservation of this locus in natural *P. falciparum* populations, a recently described population of 714 whole genome sequenced parasites from Africa and South East Asia [15] was screened for the presence of the PFGA01_060022400 and PF3D7_0617400. PFGA01_060022400 was detected at full length and > 95% identity in 36 of the 714 fully sequenced isolates. PFGA01_060022400 was detected at equal frequency in parasites from Africa and Asia clearly confirming the conservation of this locus in natural *P. falciparum* populations on different continents (Table 2). Interestingly, the conservation of PFGA01_060022400 was in the same order of magnitude as the conservation of PFDd2_070015900, a *var* gene located in the *P. falciparum chloroquine resistance transporter* (*pfcrt)* associated genetic sweep on chromosome 7, that was detected in 33 isolates of the global *P. falciparum* population.

**Table 2:** Conservation of PFGA01_060022400 in 714 isolates from Africa and Asia

Country of origin	Analysed isolates	PFGA01_060022400 > 3500 bp
The Gambia	60	9
Malawi	60	8
Laos	60	6
Cambodia	60	6
Mali	60	4
Ghana	60	4
Congo	60	3
Vietnam	59	3
Thailand	60	3
Guinea	60	2
Kenya	55	1

In contrast to PFGA01_060022400, the full length allele of PF3D7_0617400 was only detected in 3 isolates. However, when fragments between 3,5 kb and 500 bp were analysed the prevalence of the two alleles was similar and increased from 48 to 139 isolates (Table 3).

**Table 3:** Conservation of fragments from 5000 to 500 bp in 714 isolates from Africa and Asia

Size of fragment	PFGA01_060022400	PF3D7_0617400
> 5000 bp	36	3
> 4000 bp	42	3
> 3500 bp	48	41
> 3000 bp	50	44
> 2500 bp	51	45
> 2000 bp	76	108
> 1500 bp	92	129
> 1000 bp	94	131
> 500 bp	100	139

**Fig. 7:**
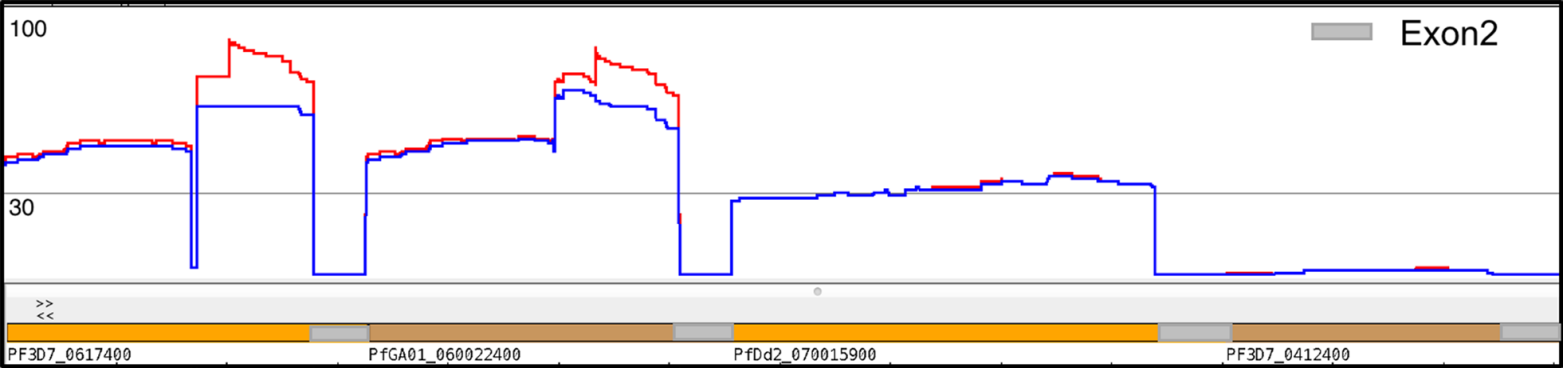
Exon 1 conservation of PF3D7_0617400, PFGA01_060022400, PFDd2_070015900 and PFD3D7_0412400 in a global population of parasites. The amount of sharing of 4 *var* genes among a population of 714 parasites from Africa and Asia [15]. Blue parts correspond to fragments > 2000 bp, red parts correspond to fragments > 500 bp. The y axis shows the number of isolates carrying the respective fragments. Note that the second part of exon I is conserved to a higher degree than the first part. The small insertion that marks the difference between PF3D7_0617400 and PFGA01_060022400 can be appreciated. Note that PFDd2_070015900 is conserved along the entire exon1, consistent with a location within the *pfcrt* genetic sweep. PFD3D7_0412400 is shown as an example of a nonconserved UpsC 3D7 *var* gene. Exon 2 sequences were excluded from the analysis

Next, the conservation of the different parts of exon 1 of PFGA01_060022400 was analysed. This revealed that the first part of exon 1, corresponding to the DBLa0.21, CIDRa2.1 and DBLb4 (0-approximatley 3700 bp) were less conserved than the second part of exon 1 (approximately 3800–5800 bp) corresponding to the CIDRb1 and DBLd1 (Fig. 7). In contrast, a *var* gene (PfDd2_070015900) located in the *pfcrt* associated genetic sweep on chromosome 7 was equally conserved along the entire exon1 (Fig. 7). Furthermore, a SNP analysis in 19 isolates carrying PFGA01_060022400 showed no evidence of a genetic sweep in the chromosomal area surrounding the central cluster on chromosome 6 (Additional file 4: Fig. S2).

An analysis of conservation of all 3D7 *var* genes revealed that, except for the pseudogene *var1csa*, PF3D7_0617400 was the most conserved 3D7 *var* gene, although fragments of several other *var* genes were also conserved to a lesser degree in the global population of *P. falciparum* parasites (Additional file 5: Fig. S3). Together the data showed that individual parts of PFGA01_060022400/PF3D7_0617400 and other *var* genes exhibited less recombination than the remainder of the gene family.

### Comparative analysis of microsatellite (MS) and *var2csa* genetic diversity

To further investigate the hypothesis that recombination of individual shorter *var* sequences might be limited, the genetic diversity of 57 microsatellite markers (average length approximately 160 bp) and two short *var2csa* fragments (156 bp and 141 bp length) was compared in the original population of 10 field isolates.

**Fig. 8:**
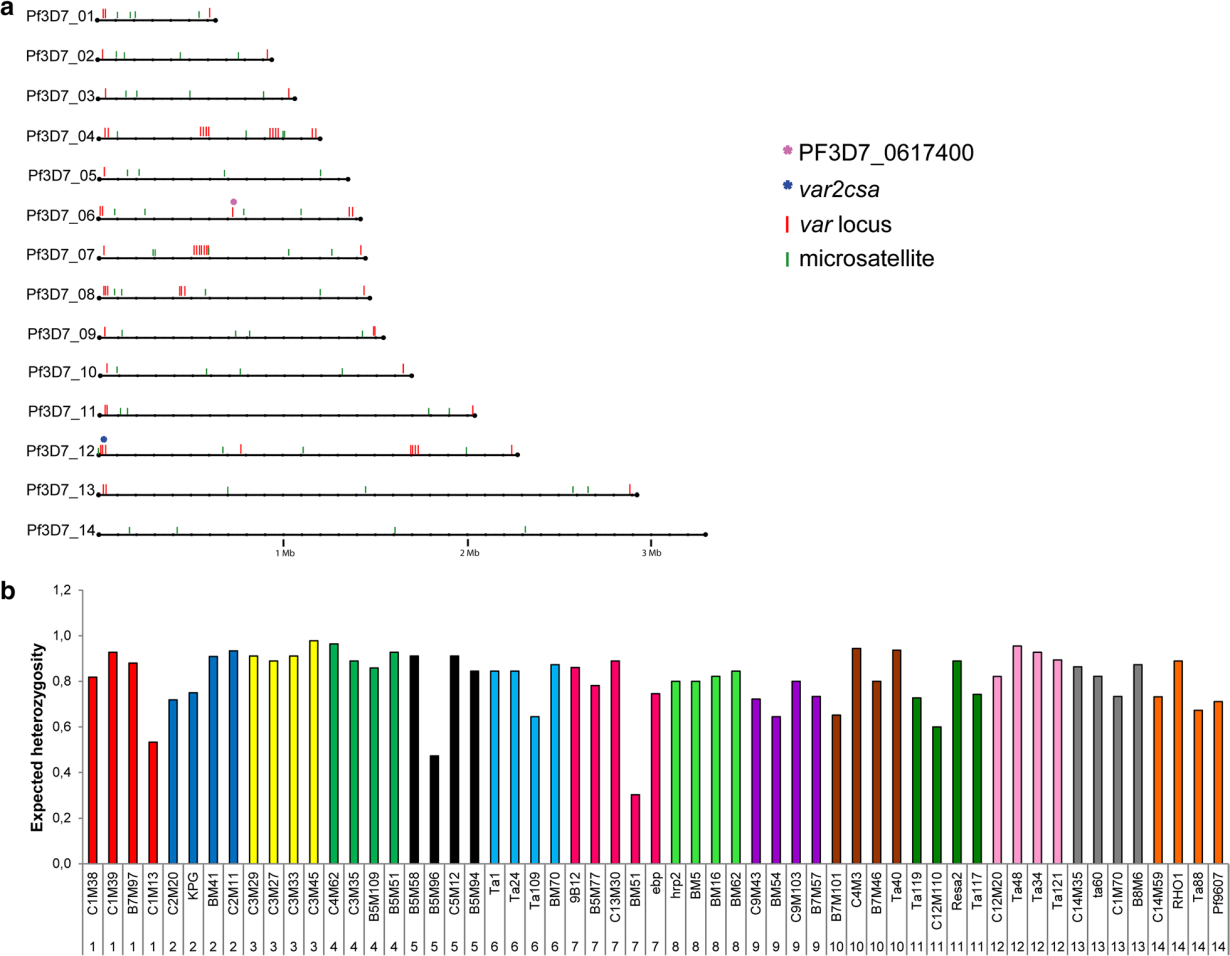
Microsatellite position and genetic diversity **a** 14 chromosomes of *P. falciparum* with positions of *var* genes indicated in red and positions of microsatellites indicated in green. **b** Observed H_e_ of microsatellite loci on all 14 chromosomes of *P. falciparum* across all field isolates. 0=no diversity, 1= max. diversity

A total of 57 MS distributed across the 14 *P. falciparum* chromosomes (three to four MS per chromosome) (Fig. 8a) were typed for each field isolate. Allele analysis of the 57 MS across the 10 field isolates revealed the presence of a unique allele in each strain at the vast majority of MS. Fragment length diversity was therefore high across all microsatellites with an expected heterozygosity (H_e_) ranging between 0,53 and 0,98 and an average H_e_ across all microsatellites of 0.8 (Fig. 8b) (Additional file 2: Table S2).

**Fig. 9:**
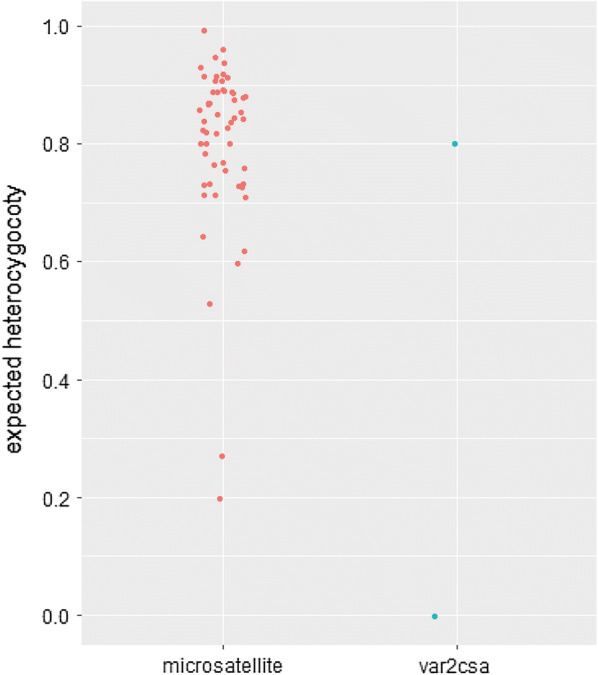
Scatterplot showing the H_e_ of the 57 microsatellites versus the two H_e_ of fragment (I) and (II) of *var2csa.* Note that the H_e_ of the fragment I of *var2csa* is 0 and thus highly conserved. In contrast the H_e_ of fragment II is 0,8 and thus in the same range as the H_e_ of the MS markers

The expected heterozygosity (H_e_) of *var2csa* was calculated for two fragments, a highly conserved (bp 3402–3558) (156 bp) (fragment I) as well as a variable region (bp 2664–2805) (141 bp) (fragment II). Fragment I had the same length and sequence in all 10 isolates and the H_e_ was 0. Fragment II differed in length and sequence in all field isolates and had a H_e_ of 0.86. Sequence analysis of this fragment revealed deletions, insertion as well as single base pair substitutions as the most common differences (data not shown). Dot plot comparison of the 57 MS H_e_s and the two *var2csa* fragments H_e_s showed that fragment II was as diverse as the majority of the MS whereas fragment I was highly conserved (Fig. 9).

## Discussion

In this work, a conserved *var* gene PFGA01_060022400/PF3D7_0617400 (previously annotated as MAL6P1.252 /PFF0845c) is identified in parasites from Africa and Asia. PFGA01_060022400/ PF3D7_0617400 is located in the central cluster of chromosome 6. The predicted PfEMP1 domain structure of PFGA01_060022400/PF3D7_0617400 consists of a DBLa0.21, a CIDRa2.1, a DBLb4, a CIDRb1 and aDBLd1 domain. The only difference between the 3D7 and the field isolate allele is a small insertion of approximately 190 bp located between the DBLb4 and CIDRb1.

The CIDR a2.1 [31] of PFGA01_060022400/PF3D7_0617400 possesses the recently described hydrophobic pocket that is responsible for PfEMP1 binding to the CD36 receptor [18]. Consistent with this, NF54 parasites transcribing PF3D7_0617400 bound efficiently to CD36 receptors on human melanoma cells, showing that the expressed PfEMP1 exhibits the promiscuous CD36 binding phenotype. Although the hydrophobic pocket is present in virtually all CIDRa2-6, the sequence similarity is generally very low, as is the overall sequence similarity across the global population of CIDRa2-6 [32]. This raises the question which binding phenotype might be conferred by the remainder of the PFGA01_060022400/ PF3D7_0617400 domains.

Metwally et al. [31] conducted a comprehensive cytoadhesion analysis of the 3D7 laboratory strain on CHO-745 WT cells and in CHO-745 cells expressing recombinant CD36, Intracellular Adhesion Molecule 1 (ICAM) 1, P-selectin, E-selectin, CD9 and CD151. 3D7 parasites showed strong upregulation of PF3D7_0617400 transcription after selection on all cell types. The strongest upregulation was seen after binding selection on Chinese Hamster Ovary (CHO) wild type (WT) cells with PF3D7_0617400 being the only significantly upregulated *var* gene (84% of the total *var* gene signal). Together these data suggest that PF3D7_0617400 is able to bind to a yet unidentified receptor on CHO WT cells as well as CD36, ICAM, P-selectin, E-selectin, CD9 and CD15. Synergistic binding to multiple receptors has [33] been shown to confer more efficient binding providing a potential explanation why PF3D7_0617400/ PFGA01_060022400 might confer a selective advantage.

The field isolate clone MOA C3 transcribed PFGA01_060022400 in a very stable fashion yet it was not possible to increase the low binding capacity of the strain to human melanoma cells expressing recombinant CD36. The MOA parasite line was originally obtained from a chronic asymptomatic infection that lasted for more than 90 days. The MOA parasites had therefore been under strong selective pressures prior to tissue culture adaptation. A previous phenotypic analysis of 19 clones from the MOA bulk culture showed that immune recognition of MOA clones did not correlate with *var* gene transcription [20]. Here, these observations are extended by phenotypic profiling of an NF54 and a MOA clone transcribing the same *var* gene. CD36 selected NF54 A3 and MOA C3 parasites exhibited a marked difference in surface recognition signal and CD36 binding, yet the corresponding PfEMP1 was detected by immunofluorescence assay (IFA) in both cell lines. This suggests a difference in PfEMP1 display between the two cell lines. Several investigations have reported that PfEMP1 expression is influenced by semi-immunity [20, 34–36] and Hoo et al. [37] have shown recently that the duration of replication in the human host has a strong impact on the *P. falciparum* transcriptome. It is thus tempting to hypothesize that the prolonged intra-host replication of the MOA parasites selected for parasites with reduced PfEMP1 display to allow the submicroscopic parasitaemia of chronic infections. It is conceivable that expression of a PfEMP1 variant with synergistic binding to multiple receptors provides a selective advantage during chronic infections. Future investigations with culture adapted parasites from semi-immune individuals are necessary to further address this question.

PFGA01_060022400 was identified in 37 isolates of a recently characterized global population of 714 fully sequenced isolates. A previous analysis of this population had identified several *var* genes with fragments > 3.5 kb that were shared at 99% percent identity in up to 55 isolates. These genes are located on chromosomes 7, 4, and 8 in the areas of drug resistance associated selective sweeps. An additional conserved *var* gene was located in a telomeric genetic sweep of chromosome 6. In contrast the parasite isolates carrying PFGA01_060022400/ PF3D7_0617400 showed no evidence of a genetic sweep in chromosomal areas flanking the conserved locus in the central cluster of chromosome 6. This suggests that PFGA01_060022400/PF3D7_0617400 might provide some independent “fitness advantage”. The population analysis also showed that the DBLb4 and CIDRb1 sequences were present in more isolates than the DBLa0.21, CIDRa2.1, DBLb4, CIDRb1 sequences, indicating that fragments of the gene are conserved to different degrees in the global *P. falciparum* population, suggesting that individual sequence blocs might be under “positive selection”.

To further evaluate this hypothesis, the genetic diversity of a highly variable and highly conserved part of the DBL2x domain of the *var2csa* gene and the genetic diversity of 57 MS [26] were compared in the original population of 10 field isolates. As expected the genetic diversity of MS was high (0.76–0.8) and in the range of previously reported values for MS genetic diversity [27, 38–40]. Strikingly, the variable DBL2x part exhibited the same average genetic diversity (H_e_ = 0.8) as the MS, suggesting that sequence variation in these parts of the gene have no deleterious effect on parasite fitness. In contrast the genetic diversity of the conserved DBL2x fragment was 0 and indeed the sequence was completely identical across all isolates. These data are consistent with a recent analysis of the *var2csa* gene in a global population of >2000 isolates that showed a high sequence diversity along the DBL2x but a high degree of conservation in the area corresponding to the conserved fragment [41].

While *var2csa* is present in every parasite, the full length PFGA01_060022400/ PF3D7_0617400 was only detected in approximately 5% of field isolates. PFGA01_060022400 /PF3D7_0617400 is located in the central cluster of chromosome 6 and belongs to the UpsC subclass of *var* genes. UpsC *var* genes have been shown to be preferentially transcribed during long term in vitro culture and during chronic asymptomatic infections [19, 22, 42]. The binding phenotype of PFGA01_060022400 /PF3D7_0617400 may, therefore, be important to establish chronic asymptomatic infections. If receptor binding is indeed responsible for the conservation of PFGA01_060022400 /PF3D7_0617400 this would imply that other *var* genes must confer the same binding phenotype. The recent global analysis of the *var* gene family by Otto et al. [15] showed that UpsC genes are the most conserved among the *var* gene classes which could be consistent with a common binding phenotype mediated by UpsC *var* genes. In summary, the data reported here suggests that individual PfEMP1 binding phenotypes may limit the sequence diversity of individual members of the *var* gene family.

## Conclusion

In this study, a conserved *var* gene on chromosome 6 is identified in parasites from Africa and Asia. This indicates that some *var* loci appear to be under purifying selection because they provide a selective advantage. The conserved *var* gene binds to multiple receptors, a phenotype that might increase parasite fitness during chronic infections. Interestingly, direct phenotypic comparison of a laboratory and a field isolate from a chronic infection expressing the locus showed marked differences in receptor binding suggesting a difference in PfEMP1 display.

## Supplementary information

**Additional file 1: Table S1.** Exon 1 Primer pairs designed for targeted Sanger sequencing of PF3D7_0617400.

**Additional file 2: Table S2.** base pair length and H_e_ of 57 MS across the population of field isolates.

**Additional file 3: Fig. S1.** Quantitative transcriptional analysis with RTPCR. a) Transcriptional profile of the *var* gene family after CD36 binding selection of NF54 A3 shows a strong increase in transcriptional signal of PFD_0617400. b) Transcriptional profile of MOA C3 after CD36 binding selection of NF54 A3 shows no change in transcriptional signal of PFD_0617400.

**Additional file 4: Fig. S2.** SNP analysis of chromosome 6 of 19 parasites carrying PFGA01_060022400. The area of chromosome 6 flanking the central cluster is shown. The position of PFGA01_060022400 is depicted by the vertical red line. SNPs are indicated by bars of different colour. There is no evidence of genetic sweep in the areas flanking the locus.

**Additional file 5: Fig. S3.** Analysis of 3D7 *var* gene conservation within the global population of 714 parasites. Fragments of > 3000 bp that are conserved within the global population are depicted. The red square identifies PF3D7_0617400.

## Data Availability

The datasets supporting the conclusions of this article are included within the article and its additional files.

## Abbreviations

CD: Cluster of differentiation
iRBCs: Infected red blood cells
PfEMP1: *Plasmodium falciparum* erythrocyte membrane protein 1
EPCR: Endothelial protein C receptor
Ups: Upstream
DC: Domain cassette
DBL: Duffy binding like
CIDR: Cysteine rich interdomain
CSA: Chondroitin sulfate A
PCR: Polymerase chain reaction
ORF: Open reading frame
qPCR: Quantitative PCR
*pfcrt*: *Plasmodium falciparum chloroquine resistance transporter*
MS: Microsatellite
H_e_: Expected heterozygosity
ICAM 1: Intracellular adhesion molecule 1
CHO: Chinese Hamster Ovary
WT: Wilde type
IFA: Immunofluorescence assay
MFI: Mean fluorescence intensity
