# Supplementary material for: Identification of a conserved var gene in different Plasmodium falciparum strains
Source: Malar J. 2020 May 29;19:194. doi: 10.1186/s12936-020-03257-x (PMC7260770; doi:10.1186/s12936-020-03257-x)
Supplement: Supplementary file 1 — Additional file 1: Table S1. Exon 1 Primer pairs designed for targeted Sanger sequencing of PF3D7_0617400. [file 12936_2020_3257_MOESM1_ESM.docx]

| **Primer** | **Forward seq. 5'3'** | **Reverse seq. 5'3'** |
| --- | --- | --- |
| P0 | TGCCAAGGACCTTTTGGAA | GGTAATATTAATAACACAGAT |
| P39 ^1^ | ATTTGTCGCACATGAAGGAA | TACAGCATTGGC |
| P00 | TCTGTTTCTTGGTTATACC | GGTGATTCTTCAGATT |
| P0.1 | CAAGATCCTGCAAAATCTCA | CTTTAGAGAAATGTCGTGGA |
| P1 | GATGAATGGAGCGAAGAA | AAAAATTCTAGTTTTTCGTTA |
| Primer 1.1 | TAAAAGAAAGAGAACCTAC | TCGAAACATTTTCTCATAC |
| P2 | GCGAACCTTGCCCTTGGTGT | CATTCTATAGAATCATCTAACA |
| P1.1.1 | GCATATTAATCATGATATTTGG | CGACGAAGAAGAAACGGATA |
| Primer 2.1 | GTATACGGGGTTGTAAAAAG | GCTTATGGAATAGAAAAATC |
| P3 | CACGGAATTATAAGAGATAC | CCATGACATGGTCCTTGCGGA |
| P2.1.1 | GAAACCCATAATGATGAAAA | CAGATTTCATAAAAGAGAAG |
| Primer 3.1 | GAAACCTGGGAACCAGAT | GATTGGTGGGAGGCAAACA |
| P3.1.1 | GACAGAACACTTAATGGTAT | CAAGTTGTAAGAAAAAGGGT |
| P4 | GAGCTATGACGTGTGCAACA | ACTTCGCATTCACCTACACG |
| P4.1.1 | CTCAAAGATTACGTTGGATG | CCACGAAAAATTCCCCAATT |
| Primer 4.1 | CGAAGAAGCATGTGCTTGT | GATTGCTTTGTGAAAAGTGC |
| P5.1.1 | GCACCAGAAAAGAAGAAAG | CAAATGTTCTACACATTGG |
| P5 | TTGAGACGTTTTTCCTATGGCAT | GTCGCCGCGCGATAGGGCGCCA |
| P6.1.1 | CAAAAGGAAAAGAAACCACA | GTGTCACACTTAAAGAAGAT |
| Primer 5.1 | TGGTGGGAACAACATGGTAA | GCGTGTGATCGAACAAATAC |
| P7.1.1 | GAATGATTTGCGCTTTAACC | CAATTTTGCGGAACACCAG |
| P6 | GCTTCTGCTGATTTAGAAGGT | GCCGTAATCTTTCCTGTTGTGCAA |
| P8.1.1 | CAAGAAAAGGCATATGGTG | GTCGTAATGTATGTGGTTATA |
| Primer 6.1 | GAAGTTGTTATGCGTGTTAG | TGGCTATGGAGAAAATTAC |
| P7 F | CACATTGGGTAGATAATTTT | CAATCCACAAGAAAAATGTGA |
| P8F | CCAACTTTAGATGCTTTCG | CAAAAGGCAACCGAAAAAAC |
| P9F | CCAGAAATTTGTAAAGATGT | GTTTCGTGTATATGTATGTG |

S1 Table: Primer set for PFF0845c Exon1

^1^Salanti A, Staalsoe T, Lavstsen T, Jensen ATR, Sowa MPK, Arnot DE, et al. Selective upregulation of a single distinctly structured var gene in chondroitin sulphate A-adhering *Plasmodium falciparum* involved in pregnancy-associated malaria. Mol Microbiol. 2003;49:179–91.
