# Supplementary material for: Identification of a conserved var gene in different Plasmodium falciparum strains
Source: Malar J. 2020 May 29;19:194. doi: 10.1186/s12936-020-03257-x (PMC7260770; doi:10.1186/s12936-020-03257-x)
Supplement: Supplementary file 2 — Additional file 2: Table S2. base pair length and He of 57 MS across the population of field isolates. [file 12936_2020_3257_MOESM2_ESM.docx]

| MS | 5798 Togo | Sanyang Gambia | 3256 Togo/Ghana | 12259 Cameroun | 5259 Kongo | 6210 Sudan | 3324 Kenya | MOA Gabon | 5420 Kenya | 12480 Kenya | **He** |
| --- | --- | --- | --- | --- | --- | --- | --- | --- | --- | --- | --- |
| C1M38 | 158 | 173 | 163 | 165 | 165 | 162 | 162 | 162 | 171 | 171 | **0,82** |
| C1M39 | 122 | 172 | 170 | 170 | 113 | 216 / 293 | 137 | 148 | 183 | 135 | **0,93** |
| B7M97 | 200 / 194 | 200 | 190 | 185 | 218 | 193 | 191 | 185 | 196 / 193 | 196 | **0,88** |
| C1M13 | 136 | 134 | 130 | 134 | 130 | 130 | 136 | 128 | 128 | 128 | **0,53** |
| C2M20 | 123 | 137 | 137 | 119 | 121 | 141 | 135 | 123 | 121 | 121 | **0,72** |
| KPG | 163 | 167 | 151 | no peak | 167 | no peak | 163 | 167 | 165 | 149 | **0,75** |
| BM41 | 155 / 161 | 151 | 157 | 177 | 154 | 171 | 146 | 154 | 173 | 157 | **0,91** |
| C2M11 | 133 | 123 | 99 | 129 | 131 | 129 | 139 | 116 | 110 | 121 | **0,93** |
| C3M29 | 184 | 185 | 157 | 174 | 165 | 160 | 170 | 181 | 173 | 184 | **0,91** |
| C3M27 | 139 | 158 | 164 | 148 | 166 | 145 | 154 | 148 | 141 | 150 | **0,89** |
| C3M33 | 115 | 134 | 137 | 115 | 101 | 119 | 111 | 117 | 95 | 95 | **0,91** |
| C3M45 | 137 | 156 | 85 | no peak | 150 /167 | 114 | 177 | 144 | 144 | 131 | **0,98** |
| C4M62 | 241 / 226 | 190 | 149 | 253 | 241 | 221 | 236 | 224 | 223 | 257 | **0,96** |
| C3M35 | 207 | 186 | 191 | 194 | 183 | 200 | 188 | 206 | 191 | 191 | **0,89** |
| B5M109 | 176 / 121 | 176 | 159 | 139 | 123 | 176 | 176 | 120 | 134 /159 | 159/ 139 | **0,86** |
| B5M51 | 221 / 243 | 237 | 214 | 224 | 249 | 224 | 220 | 214 | 227 | 227 | **0,93** |
| B5M58 | 191 | 194 | 193 | 202 | 163 | 204 | 167 | 202 | 209 | 205 | **0,91** |
| B5M96 | 172 / 177 | 168 | 177 | 173 | 177 | 177 | 178 | 177 | 177 | 177 | **0,47** |
| C5M12 | 151/ 170 | 157 | 177 | 178 | 179 | 161 | 162 | 168 | 171 | 151 | **0,91** |
| B5M94 | 142 | 138 | 138 | 149 | 179 | 147 | 147 | 157 | 142 | 142 | **0,84** |
| Ta1 | 176 | 179 | 173 | 178 | 202 | 170 | 176 | 194 | 173 | 176 | **0,84** |
| Ta24 | 187 | 193 | 194 | 198 | 203 | 198 | 211 | 187 | 187 | 198 | **0,84** |
| Ta109 | 168 | 177 | 177 | 174 | 177 | 177 | 174 | 186 | 177 | 177 | **0,64** |
| BM70 | 173 | 193 | 193 | 183 / 206 | 189 | 197 | 175 | 191 | 175 | 189 | **0,87** |
| 9B12 | 167 / 171 | 160 | 163 | 160 | no DNA | 165 | 169 |  | 165 | 158 | **0,86** |
| B5M77 | 141 | 141 | 150 | 150 | 143 | 143 | 131 | 154 | 143 | 145 / 154 | **0,78** |
| C13M30 | 187 | 203 | 193 | 193 | 202 | 197 | 201 | 207 | 223 | 185 | **0,89** |
| BM51 | 140 | 140 | 140 | 140 | 139 | 140 | 140 | 140 | 134 /140 | 134 / 140 | **0,30** |
| ebp | 134 | 136 | 134 | 134 | 139 | 138 | 141 | 136 | 138 | 147 / 149 | **0,75** |
| hrp2 | 167 | 167 | 178 | 182 | 175 | 189 | 173 | 173 | 175 | 169 | **0,80** |
| BM5 | 134 | 136 | 124 | 133 | 138 | 158 | 138 | 136 | 136 | 139 | **0,80** |
| BM16 | 147 | 157 | 153 | 147 | 142 | 142 | 149 | 144 | 157 | 144 | **0,82** |
| BM62 | 166 | 170 | 164 | 162 | 158 | 168 | 164 | 168 | 158 | 160 | **0,84** |
| C9M43 | 130 /138 | 126 | 124 | 128 | 128 | 130 | 132 | 134 | 132 | 128 | **0,72** |
| BM54 | 165 | 158 | 164 | 167 | 167 | 164 | 163 | 185 | 164 | 164 | **0,64** |
| C9M103 | 149 | 149 | 145 | 153 | 136 | 138 | 149 | 145 | 144 / 152 | 145 | **0,80** |
| B7M57 | 219 | 227 | 220 | 220 | 219 | 223 | 229 | 223 | 223 | 229 | **0,73** |
| B7M101 | 204 | 204 | 204 | 204 | 196 | 229 | 204 / 185 | 204 | 229 | 185/ 204 | **0,65** |
| C4M3 | 173 | 178 | 150 | 171 | 129 | 163 | 190 | 181 | no peak | 183 | **0,94** |
| B7M46 | 187 | 150 | 169 | 161 | 160 | 178 | 169 | 161 | 170 | 160 | **0,80** |
| Ta40 | 212/ 125 | 191 | 203 | 215 | 218 | 231 | 216 | 215 | 191 / 218 | 209/ 222 | **0,94** |
| Ta119 | 228 / 235 | 235 | 250 | 250 | 250 | 235 | 238 | 251 | 250 | 250/ 219 | **0,73** |
| C12M110 | 157 | no peak | 157 | no peak | 129 | 157 | no peak | no Peak | 154 | 156 | **0,60** |
| Resa2 | 101 | 99 | 91 | 101 | 102 | 96 | 113 | 105 | 97 | 105 | **0,89** |
| Ta117 | 169 / 175 | 180 | 168 | 180 | 175 | 186 | 175 | 181 | 180 | 192 / 175 | **0,74** |
| C12M30 | 114 | 176/ 123 | 209 | 176 | no peak | 210 | 115 | no Peak | no peak | 176 | **0,82** |
| Ta48 | 270 | 298 | 313 | 256 | 270 | 256 | 262 | 276 | 290/ 303 | 276/ 313 | **0,95** |
| Ta34 | 105 | 116 | 144 | 139 | 105 | 106 | 133 | 135 | 126 | 111/ 151 | **0,93** |
| Ta121 | 155 / 167 | 152 | 157 | 158 | 167 | 161 | 155 | 158 | 158 | 161 | **0,89** |
| C14M35 | 181 / 204 | 166 | 197 | 181 | 166 /163 | 166 | 166 | 189 | 180 | 174 | **0,86** |
| ta60 | 191 | 202 | 202 | 199 | 202 | 190 | 193 | 194 | 199 | 190 | **0,82** |
| C1M70 | 161 | 165 | 162 | 165 | 164 | 164 | 167 | 164 | 170 | 168 | **0,73** |
| B8M6 | 125 / 134 | 128 | 122 | 119 | 122 | 119 | 131 | 122 | 128 | 119 | **0,87** |
| C14M59 | 153 | 162 | 145 | 147 | 147 /155 | 162 | 147 | 160 | 151 | 151 | **0,73** |
| RHO1 | 195 | 218 | 216 | 214 | 194 | 194 | 203 | 199 | 201 | 208 | **0,89** |
| Ta88 | 217 | 222 | 216 | 216 | 217/ 220 | 216 | 229 | 213 | 220 | 217 | **0,67** |
| Pf9607 | 111 | 114 | 114 | 111 | 117 | 111 | 111 | 114 | 114 | 117 | **0,71** |
