# Supplementary figures and images for: Identification of a conserved var gene in different Plasmodium falciparum strains
Source: Malar J. 2020 May 29;19:194. doi: 10.1186/s12936-020-03257-x (PMC7260770; doi:10.1186/s12936-020-03257-x)

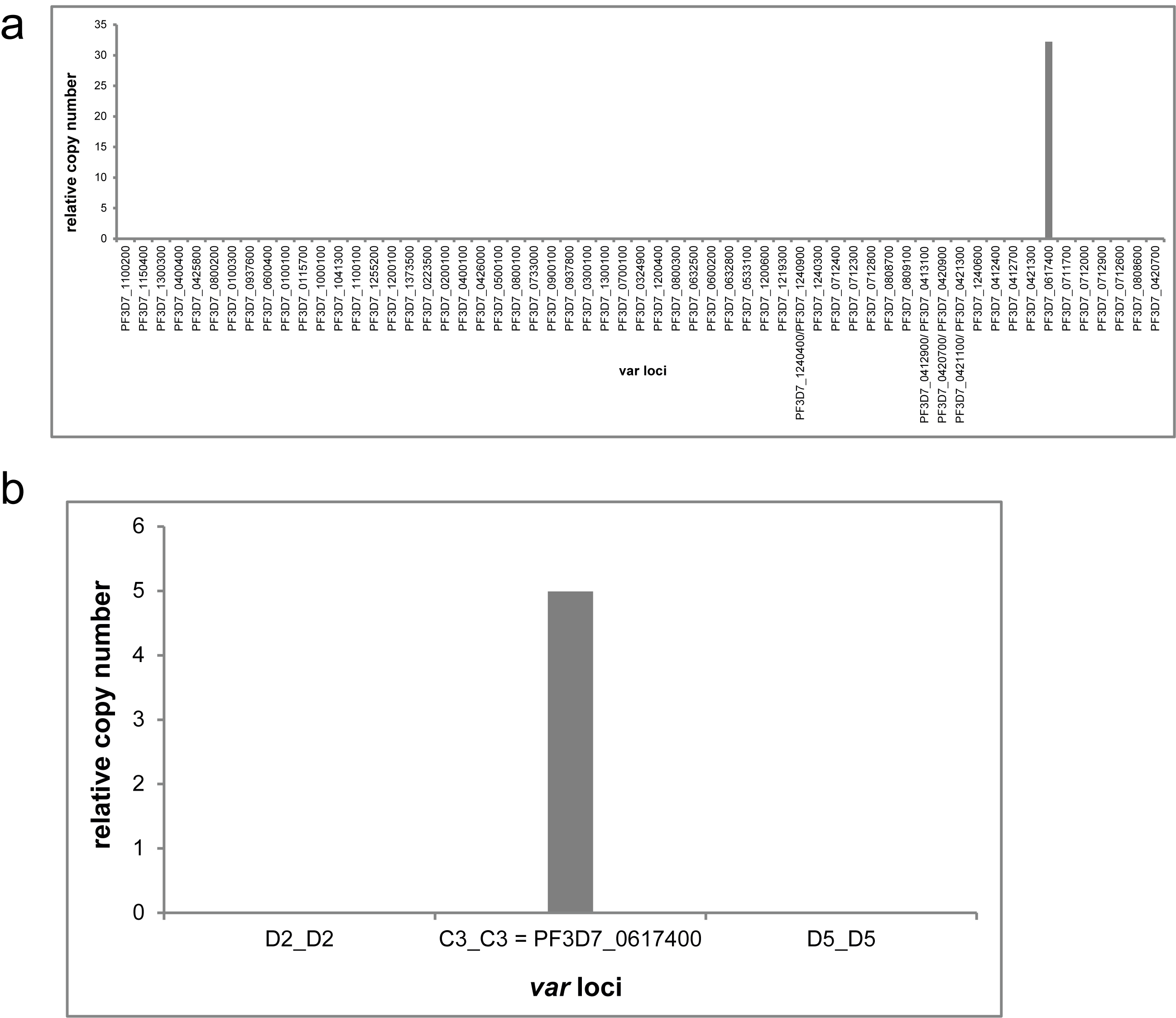

Supplement: Supplementary file 3 — Additional file 3: Fig. S1. Quantitative transcriptional analysis with RTPCR. a) Transcriptional profile of the var gene family after CD36 binding selection of NF54 A3 shows a strong increase in transcriptional signal of PFD_0617400. b) Transcriptional profile of MOA C3 after CD36 binding selection of NF54 A3 shows no change in transcriptional signal of PFD_0617400. [file 12936_2020_3257_MOESM3_ESM.tif]

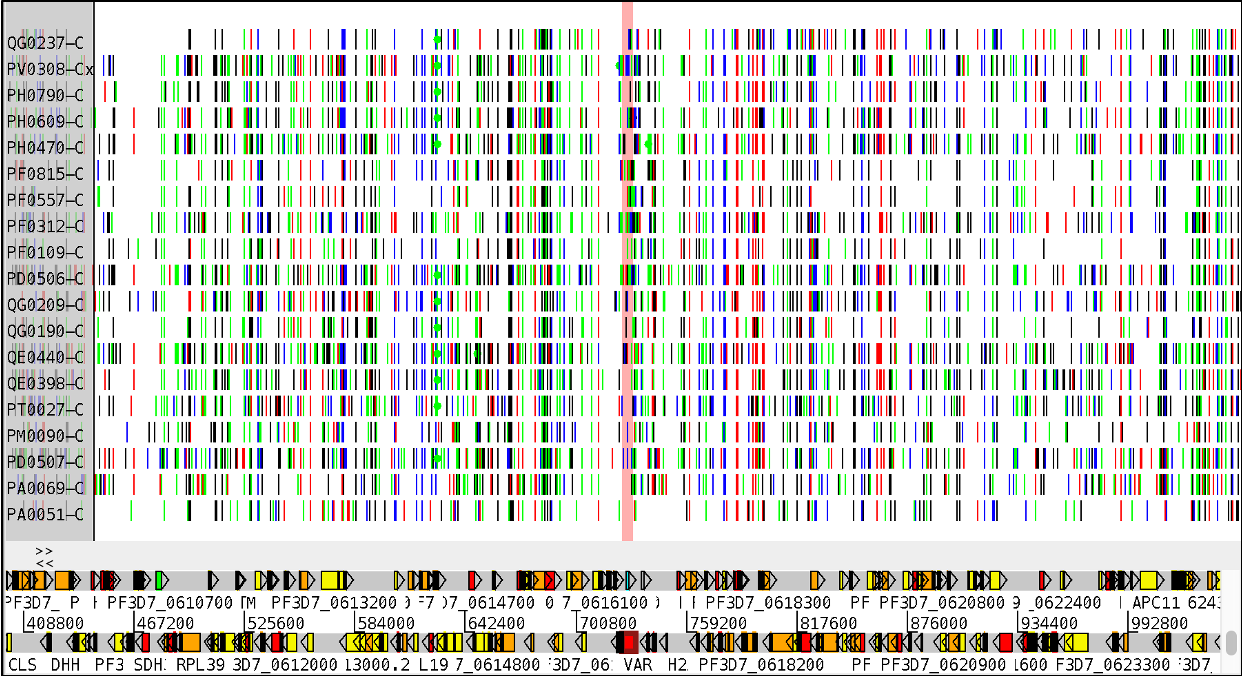

Supplement: Supplementary file 4 — Additional file 4: Fig. S2. SNP analysis of chromosome 6 of 19 parasites carrying PFGA01_060022400. The area of chromosome 6 flanking the central cluster is shown. The position of PFGA01_060022400 is depicted by the vertical red line. SNPs are indicated by bars of different colour. There is no evidence of genetic sweep in the areas flanking the locus. [file 12936_2020_3257_MOESM4_ESM.png]

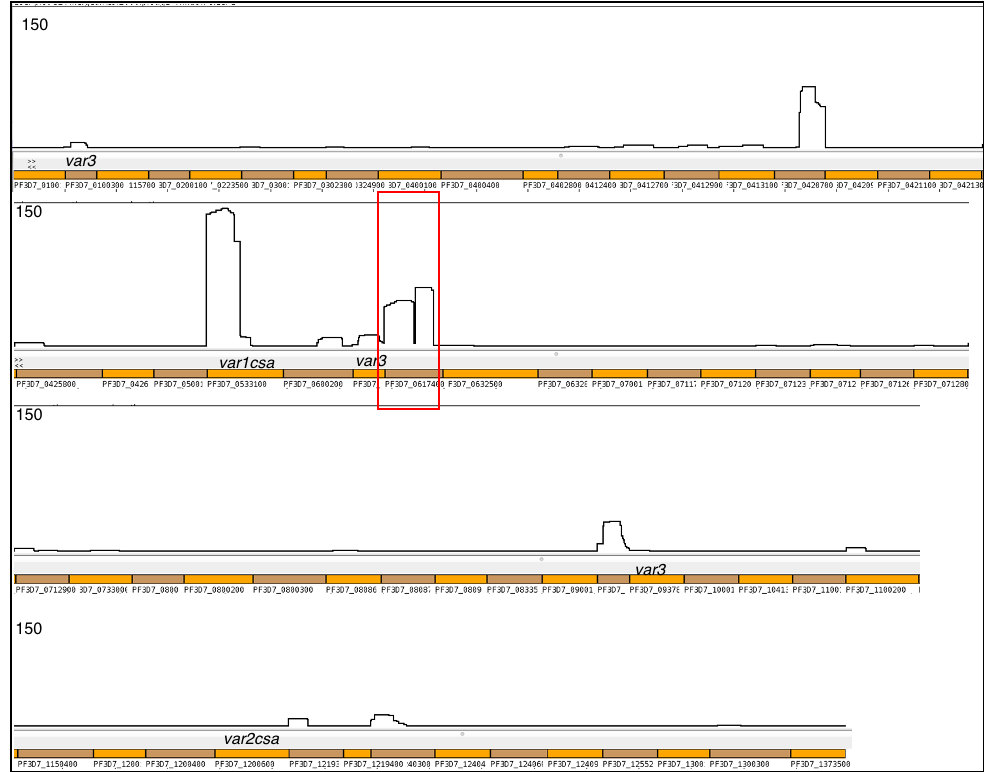

Supplement: Supplementary file 5 — Additional file 5: Fig. S3. Analysis of 3D7 var gene conservation within the global population of 714 parasites. Fragments of > 3000 bp that are conserved within the global population are depicted. The red square identifies PF3D7_0617400. [file 12936_2020_3257_MOESM5_ESM.png]
